# THE LIFESTYLE OF MEN AND WOMEN AGED 50+ IN TAIWAN: ACTIVITY ON TIME OF DAY AND THEIR CIRCADIAN RHYTHMS

**DOI:** 10.1093/geroni/igad104.3055

**Published:** 2023-12-21

**Authors:** Ching-Ju Chiu

**Affiliations:** National Cheng Kung University, Tainan City, Tainan, Taiwan (Republic of China)

## Abstract

**Background:**

To identify the association of circadian rhythms and objective measures of physical activity at a different time-zone of day, as well as examine gender differences.

**Methods:**

Participants aged 50 years and older living in the community in Taiwan were recruited. Those who suffer from dementia (cognitive dysfunction), mental illness, depression, or those taking drugs that affect daytime activities (e.g. sleeping pills, central nervous system drugs) were excluded. Activity was measured by wearable actigraphy device for at least seven days, diary, and self-reported questionnaires.

**Results:**

Among the 55 participants enrolled in the beginning, 34 (62%) meet the criteria that wore a wearable actigraphy device at least 7 completed days were analyzed. The activity in the morning was the highest among the participants. Although not statistically significant, as the age increases, the amount of activity decreases (H value=4.943, P=0.084); the amount of activity in the evening is higher for people with higher education(Z=-1.776, P=0.076); people with lower loneliness had higher activity levels (Z=-1.622, P=0.105); people with better nutritional status had higher activity (Z=-1.656, P=0.098). The amount of activity in women is positively and significantly related to regular exercise (Z=-2.653, P=0.007**), and nutrition(Z=-2.367, P=0.017*), while men are significantly related to retired status(Z=-2.132, P=0.033*).

**Conclusions:**

People who are older, retired, with chronic disease, more depression, and loneliness were associated with lower objective measure of physical activity. Determinant factors exist gender differences: while men are predicted by social variables (retired status), women are predicted by physiological variables (regular physical activity and nutritional status).

